# Some Thoughts on Materia Medica

**Published:** 1895-02

**Authors:** C. S. Schwenke

**Affiliations:** Philadelphia


### SOME THOUGHTS ON MATERIA MEDICA.

C. S. Schwenke, M. D., Philadelphia.

That which prompted the writing of this paper was a desire to go back over the old ground and revive a few of the things which make the application of the indicated remedy a success in the treatment of the sick. There will be no startling revelations, no new discoveries, nor any elaboration upon the "giant strides" of progress made in medical science. It will contain only a few homely and commonplace remarks suggested by *The Organon* and prompted by practical experience. It does not even possess the merit of originality, but rather presents the writer in the humble light of an interrogator, one curious to have many questions answered.

Why does the indicated remedy fail?

Is it because we go to extremes and either grovel, as some express it, in the tinctures or airily soar away on a potency with the thousandth or even the millionth as its exponent? They call one a mongrel and the other an ultra-homœopath, each equally foolish in the estimation of the other; opponents fighting the bitter war of ridicule and accomplishing nothing but personal enmity. Is any progress made by pursuing such a course? Is it not about time that we at least flatter ourselves with a degree of intelligence which will lift us above such craftiness? The indignant potentist speaks of his compeer sinking into the mire of tinctures, fluid extracts, and alkaloids until he becomes a skeptic and materialist, while the alkaloid man is jocosely horrified at the intrepidity of his infinitesimal friend toying with the dynamic derangement of the millionths.

Does this savor of progress? Just as long as we abstain from the friendly discussion of the potency of our remedies just so long will we retrogress in materia medica. The strength of a remedy is almost if not quite as important as its selection. If too strong or too crude the case may at once be spoiled by aggravation. If it is too highly diluted, doubt of its being the indicated remedy, by its inefficiency to produce any result may lead to the choice of another. Failure in each instance attributable alone to the strength in which the remedy was employed. Instead of going to extremes, would not a medium course appear more rational where remedies varying in dilution from the third to the thirtieth were used? If the physician employing the very low dilutions and tinctures should adopt such a course he might find that necessity would make of him a more accurate prescriber, while the high dilutionist, by pursuing a similar course, might save much wear and tear on his imagination.

Would not much be gained in our acquirement of the homœopathic materia medica if cases, even the most commonplace, were accurately reported, giving a careful delineation of the symptoms together with a differentiation of the remedies suitable to the case, their dilutions, and the frequency with which they were repeated? By so doing would we not in time become better satisfied with our materia medica, find in it fewer inaccuracies, and grow far better qualified to treat the sick?

When a remedy is indicated in a case of sickness no one patient manifests all of the symptoms true of the remedy. It is usually five or six and sometimes less that favor the selection. Largely because of this much fault has been found with the apparent superabundance of material furnished by our materia medica. Let us select Arsenicum by way of illustration. Each time it is called for in different patients it may be found indicated by different sets of symptoms true of Ars. Select a materia medica—Hering's condensed is convenient—for the purpose, and record all of the symptoms in the different cases cured by Arsenicum by placing a star alongside of the symptoms found in the book, and in time a star will be found alongside each symptom printed, plus a number of extra symptoms on the margins of the pages. The characteristic symptoms will be noticed by the large number of stars attached to them. This is one way of studying materia medica by which we may get the individuality of the remedy. There is no question about the amount of labor involved. It requires an enormous amount of work to gain a proper understanding of the homœopathic materia medica, but with it goes a compensation in ratio with the ability to cure the sick.

A source of common error and failure to procure results with the indicated remedy lies in the fact that the whole power of the remedy is not understood. A remedy may be correctly applied to a case and if a new symptom appears it is too often regarded as a complication while it is only a part of the whole and covered by the same remedy. Failure to appreciate this fact is invariably disastrous to the patient. This may be illustrated by any remedy in any form of disease. Let us suppose a form of enteric fever calling for Hyoscyamus, the selection having been made by a few characteristic symptoms such as the following, trembling and twitching of the muscles. Farrington regarded this as a necessary symptom of Hyosc. I have never seen a case calling for Hyosc. where this symptom was absent. In taking the pulse you are impressed by the twitching of the tendons under the fingers.

The patient imagines he sees people who are not present and who have not been present. He mixes the identity of those present, calling one by the name of another, and often regarding a personal friend as a stranger and *vice versa.* At times he may refuse the medicine, fearing it is poison. He may be lying quietly in bed when suddenly, without any warning, he will jump from the bed, and may strike these about him in his endeavor to escape. Patient seems terrified. He may be partially aroused. In this partial rationality so many have spoken of the following symptoms that I have come to regard it as one quite characteristic of Hyosc.: odd little people or gnomes, about one or two feet high, are seen gamboling along the footboard of the bed or clinging to the frieze of the room, watching the patient and terrifying him. One patient would have jumped from the window to have escaped them if the nurse had not caught him about the waist and held him. This patient was promptly relieved and cured by Hyosc.$^{30}$ in water, teaspoonful every half to one and two hours. The above symptoms were clinically observed and noticeably, they are almost entirely confined to the mental sphere in order to better illustrate the statement previously made—*i. e.*, a remedy may be correctly applied to a case, but if a new symptom appears it is too often regarded as a complication while it is only a part of the whole and covered by the same remedy, which would do all the work in a most satisfactory manner if but allowed the opportunity.

Suppose a case manifesting the symptoms enumerated above would suddenly present symptoms of a weak heart; pulse slow and small, intermittent, scarcely perceptible. If for this we use a cardiac stimulant or tonic we not only spoil the case but actually endanger the life of the patient in two ways: first, by the reaction of the stimulant possibly followed by heart-failure, or, by placing the nervous system in a condition antagonistic to the best action of the delicately-potentized remedy, thereby destroying the patient's opportunity of getting the full action of the remedy which would cure him. Turn to the heart symptoms of Hyosc. and see if the remedy selected by the mental symptoms will not meet all the requirements of the case.

Suppose the same patient becomes sleepless, first see what Hyosc. will do for you before resorting to Opium, the bromides, Chloral, or Sulphonal. Suppose in the same case, symptoms of inflammation of the brain or meninges appear, or the lungs become engorged by hypostatic congestion; the urine and fæces escape involuntarily. These are not to be regarded as complications; they are one and a part of Hyosc., and that remedy, if given singly, in a reasonable dilution and at proper intervals and allowed free and uninterrupted action, will remove them all and promptly cure the patient.

One of the most difficult of all things to acquire in the treatment of the sick is the ability to wait. This has been greatly ridiculed and justly so in some instances, but in it lies one of the greatest secrets of success. How often is a sickness prolonged or death hastened by a desire upon the part of the friends of the patient and unfortunately, to the discredit of the medical profession, by the physician himself getting confused and excited, trying to force an improvement, when, in his sober judgment, he knows that force avails nothing.

The ability to wait is born of the appreciation of improvement in a patient. Unless this is keenly observed, a change of remedies may be made to the disadvantage of the patient. We may wait for a half-hour, a day, or a week, and in some rare cases even longer, for the action of a remedy according to the acute or chronic nature of a malady. However, practical experience scarcely substantiates the theory of waiting for a month for one dose of a remedy to act. In carefully reading the reports of such cases, we are often impressed with the fact that less importance is attached to the selection of the remedy than to the extremely high dilution and single dose. It would seem that the workmanship surpassed the material.

While no endeavor is being made to underestimate the claim that one dose of the fortieth thousand will cure—in time, yet most of us feel that such ability arises from an occult power enjoyed by only a select few. If we are to judge from claims made and reports circulated, there can scarcely be a doubt about the physician prescribing one dose of the ten-millionth being equally as successful as the one who culls his medical knowledge from the different materia medica issued by the wholesale druggists of the land.

It is a question of vital importance if a far greater number of patients have not been killed outright by hasty treatment than ever died by judicious waiting, as exemplified in the extreme, by the expectant treatment giving a lower mortality than empirical drugging, while the intelligent use of the indicated remedy is immeasurably superior to either.—*The Hahnemannian Monthly*, December, 1894.

